# Laboratory comparison of consumer-grade and research-established wearables for monitoring heart rate, body temperature, and physical acitivity in sub-Saharan Africa

**DOI:** 10.3389/fphys.2025.1491401

**Published:** 2025-02-13

**Authors:** Stefan Mendt, Georgi Zout, Marco Rabuffetti, Hanns-Christian Gunga, Aditi Bunker, Sandra Barteit, Martina Anna Maggioni

**Affiliations:** ^1^ Charité - Universitätsmedizin Berlin, Institute of Physiology, Center for Space Medicine and Extreme Environments Berlin, Berlin, Germany; ^2^ IRCCS Fondazione Don Carlo Gnocchi, Milano, Italy; ^3^ Heidelberg Institute of Global Health, Heidelberg University Hospital, Heidelberg University, Heidelberg, Germany; ^4^ Department of Biomedical Sciences for Health, Università degli Studi di Milano, Milano, Italy

**Keywords:** fitness tracker, accelerometer, physiological parameters, global health, heat stress, SSA

## Abstract

**Background:**

Consumer-grade wearables are becoming increasingly popular in research and in clinical contexts. These technologies hold significant promise for advancing digital medicine, particularly in remote and rural areas in low-income settings like sub-Saharan Africa, where climate change is exacerbating health risks. This study evaluates the data agreement between consumer-grade and research-established devices under standardized conditions.

**Methods:**

Twenty-two participants (11 women, 11 men) performed a structured protocol, consisting of six different activity phases (sitting, standing, and the first four stages of the classic Bruce treadmill test). We collected heart rate, (core) body temperature, step count, and energy expenditure. Each variable was simultaneously tracked by consumer-grade and established research-grade devices to evaluate the validity of the consumer-grade devices. We statistically compared the data agreement using Pearson’s correlation *r*, Lin’s concordance correlation coefficient (LCCC), Bland-Altman method, and mean absolute percentage error.

**Results:**

A good agreement was found between the wrist-worn Withings Pulse HR (consumer-grade) and the chest-worn Faros Bittium 180 in measuring heart rate while sitting, standing, and slow walking on a treadmill at a speed of 2.7 km/h (*r* ≥ 0.82, |bias| ≤ 3.1 bpm), but this decreased with increasing speed (*r* ≤ 0.33, |bias| ≤ 11.7 bpm). The agreement between the Withing device and the research-established device worn on the wrist (GENEActiv) for measuring the number of steps also decreased during the treadmill phases (first stage: *r* = 0.48, bias = 0.6 steps/min; fourth stage: *r* = 0.48, bias = 17.3 steps/min). Energy expenditure agreement between the Withings device and the indirect calorimetry method was poor during the treadmill test (|*r*| ≤ 0.29, |bias | ≥ 1.7 MET). The Tucky thermometer under the armpit (consumer-grade) and the Tcore sensor on the forehead were found to be in poor agreement in measuring (core) body temperature during resting phases (*r* ≤ 0.53, |bias| ≥ 0.8°C) and deteriorated during the treadmill test.

**Conclusion:**

The Withings device showed adequate performance for heart rate at low activity levels and step count at higher activity levels, but had limited overall accuracy. The Tucky device showed poor agreement with the Tcore in all six different activity phases. The limited accuracy of consumer-grade devices suggests caution in their use for rigorous research, but points to their potential utility in capture general physiological trends in long-term field monitoring or population-health surveillance.

## 1 Introduction

According to the Intergovernmental Panel on Climate Change, the global average annual temperature is expected to rise by 1.5°C between 2030 and 2052 (compared to pre-industrial levels) due to greenhouse gas emissions and other human activities (Masson-Delmotte et al., 2018). A rise in global temperature causes extreme weather events, which pose an increased risk to nature, the economic, and to human health (Eitelwein et al., 2024). For example, prolonged periods of unusually low rainfall (droughts) threaten food and water security, heatwaves will lead to a significant increase in temperature-related diseases and deaths, wildfires will increase air pollution, and flooding will increase crop damage and the risk of disease. The World Health Organization estimates that climate change will cause around 250,000 additional deaths per year due to malnutrition, malaria, diarrheal diseases, and heat stress alone (World Health Organization, 2023). Climate change also adversely affect the ability to work and reduce labor productivity (Kjellstrom et al., 2009), particularly in low-income regions such as sub-Saharan Africa (SSA), where subsistence agriculture is crucial for the livelihood of small rural communities (Asare-Nuamah, 2021; Ayal, 2021). For example, the simulated effects of climate change on agricultural production in the eastern and coastal regions of Kenya predicts a at least 50% rest/hour work intensity during the planting season and a up to 50% rest/hour work intensity during the maize harvesting period for the years 2050 and 2100 (Yengoh and Ardö, 2020). As smallholder farmers use a lot of human labor, an increase in environmental temperature has a considerable impact on their health. In addition to increased cardiovascular stress and impaired physical and cognitive functions, physical exertion due to labor increases the incidence of heatstroke (Bouchama et al., 2022).

Research on the effects of environmental heat-related stress on health and work ability in low- and middle-income countries primarily relies on data from hospitals, surveys, and of Health and Demographic Surveillance Systems (Diboulo et al., 2012; Egondi et al., 2012; Katiyatiya et al., 2014; Park et al., 2018; Chavaillaz et al., 2019; Frimpong et al., 2020; Barteit et al., 2023; Sapari et al., 2023). The ability to monitor physiological responses to heat stress such as heart rate, body temperature, and physical activity directly in the field using wearable devices would provide invaluable data for managing health risks in smallholder farmers and residents in SSA. Objective monitoring of physical activity has rapidly advanced in recent decades with the development of commercial and research-grade wearables. Compared to research-grade technologies, consumer-grade wearables are often lower in cost, easier to use, less obtrusive and not tied to a specific location (Dunn et al., 2018); however, these advantages often come at the expense of data accuracy.

Despite the growing use of wearables in high-income settings, there is limited research on their application in low-income, climate-vulnerable regions such as SSA (Koch et al., 2022). Recent studies have demonstrated the utility of wearable devices in low-resource settings though concerns remain about the trade-offs between affordability and accuracy (Huhn et al., 2022; Matzke et al., 2024). The present study seeks to fill this gap by comparing the accuracy of consumer-grade wearables under controlled conditions. Previous studies already dealt with comparison of different wearables measuring the same physiological parameter under controlled conditions (Nelson et al., 2016; Gillinov et al., 2017; Wahl et al., 2017; Eisenkraft et al., 2023). In this study, however, we focus on multiple physiological parameters that are relevant for assessing the environmental impact on human health and performance at individual level. For this purpose, a sample of young adults was equipped with a set of wearable devices for monitoring heart rate, body temperature, and physical acitvity (steps, energy expenditure) during rest and activity periods in a laboratory environment.

## 2 Methods

### 2.1 Study participants

We recruited young men and women for our study among medical students through advertisements on the internal Charité student’s platform and social media. Those interested were eligible for inclusion if they were between the ages of 18 and 30 and had no history of competitive training. On the other hand, interested were excluded if they had any form of cardiovascular, metabolic, and neurological diseases, or any physical impairments that would prevent participation in an incremental test on a treadmill. Following explanations of the study aim and protocol, including experimental procedures and known risks, participants provided informed written consent prior to commencing study participation. Based on a sample size calculation using the results of a comparative study between commercial trackers and a portable ECG (Godino et al., 2020), the study sample was planned with 20 participants. To ensure conclusive statistical results at the end of the study, we recruited a total of 22 participants (11 women, 11 men). Their anthropometric data were as follows: age, mean 24.0 (SD 2.4) years; body weight, mean 70.2 (SD 7.7) kg; height, mean 176 (SD 9.1) cm; body mass index, mean 22.6 (SD 1.6) kg/m^2^. The study was approved by the Ethics Committee of Charité–Universitätsmedizin Berlin (Date: 9 April 2021, EA 4/050/21).

### 2.2 Data acquisition

The research and consumer-grade wearables considered for evaluation in this study were selected from a study protocol designed for the purpose of providing scientific information on their reliability for the use in the setting of population monitoring in SSA (Barteit et al., 2021). Table 1 provides details of the consumer- and research-grade wearables in the present study.

**TABLE 1:** Overview of selected consumer-grade and research-grade wearbles.

	Consumer-grade		Research-grade		
Weareable device	Withings Pulse HR	Tucky Thermometer	Faros Bittium 180	GENEActiv	Tcore sensor with data logger headband
Company	Withings France SA, Issy-les-Moulineaux, France	e-TakesCare, Versailles, France	Bittium Corporation, Oulu, Finland	Activinsights, Kimbolton, UK	Sensor: Drägerwerk AG and Co. KGaA, Lübeck, Germany, data logger: HealthLabFunkMaster, KORA Industrie-Elektronik GmbH, Hambühren, Germany
Dimension	18 × 10 × 44 mm	84 × 27 × 7 mm	48 × 29 × 12 mm	43 × 40 × 13 mm	Sensor: 60 × 50 × 4 mm, data logger: 48 × 30 × 5 mm
Weight	45 g	8 g	13 g	28 g	Sensor: 3 g data logger: 15 g
Wear location	wrist	under armpit	3 electrodes on thorax	wrist	forehead
Sample rate	every minute for heart rate (1 Hz in workout mode), steps, energy expenditure	every minute	up to 1,000 Hz	up to 100 Hz	0.5 Hz
Internal storage	yes	no	yes	yes	yes (data logger)
Data transfer	bluetooth low energy	bluetooth low energy	USB cable	cradle with USB cable	USB cable (data logger)
Measurement features	heart rate, distance, calories, sleep	body (shell) tempereature, sleeping position monitor	1-lead electrocardiography, tri-axial accelerometer	tri-axial accelerometer light exposure, near body temperature	body (core) temperature

#### 2.2.1 Consumer-grade wearables

Withings (Withings France SA, Issy-les-Moulineaux, France): We used the Withings Pulse HR device to measure heart rate (HR), steps taken, and calories burned. Data from the internal storage was wirelessly synchronized with a mobile device via the Health Mate application.

Tucky (e-TakesCare, Versailles, France): The Tucky device, a flexible thermometer patch, was used to measure axillary temperature. The recordings were transfered directly via Bluetooth to a mobile device that used the Tucky application.

#### 2.2.2 Research-grade wearables

Faros™ (Bittium Corporation, Oulu, Finland): The Faros Bittium 180 is a gold-standard portable one-lead electrocardiography monitor. It enables long-duration beat-to-beat recordings both inside and outside hospital and healthcare facilities (Laborde et al., 2017; Hartikainen et al., 2019; Bent et al., 2020; Funston et al., 2022; Lang et al., 2022).

Tcore™ (Drägerwerk AG and Co. KGaA, Lübeck, Germany): The Tcore sensor calculates core body temperature (CBT) using a dual-sensor heat flux technology integrated into a soft sensor attached to the forehead (Werner and Gunga, 2020). Accuracy and validty of this technology is given elsewhere (Gunga et al., 2008; Mendt et al., 2017; Soehle et al., 2020; Janke et al., 2021; Engelbart et al., 2023). In this study, the sensor cable was connected to a data logger (HealthLabFunkMaster, KORA Industrie-Elektronik GmbH, Hambühren, Germany) and the data logger was integrated into a custom-made headband.

GENEActiv (Activinsights, Kimbolton, UK): We used the GENEActiv to record raw acceleration data (range ±8 g) along three orthogonal axes (x-, y- and *z*-axis). Post-processing of the tri-axial accelerometric data enables an objective assessment of physical activities (e.g., energy expenditure, step count) and sleep behavior (Scott et al., 2017; Sanders et al., 2019; Fraysse et al., 2020; Antczak et al., 2021; Jenkins et al., 2022; Hachenberger et al., 2023).

Cortex Metalyzer 3B (CORTEX Biophysik GmbH, Leipzig, Germany): The Cortex Metalyzer 3B is a spiroergometry system designed for measuring oxygen consumption and carbon dioxide production using breath-by-breath gas analysis to calculate energy expenditure (EE) via indirect calorimetry. The device was calibrated once and directly before the study for volume and gas concentrations. For gas calibration, a mixture of 15% oxygen, 5% carbon dioxide, and balance nitrogen was used.

### 2.3 Study procedure

**FIGURE 1:**
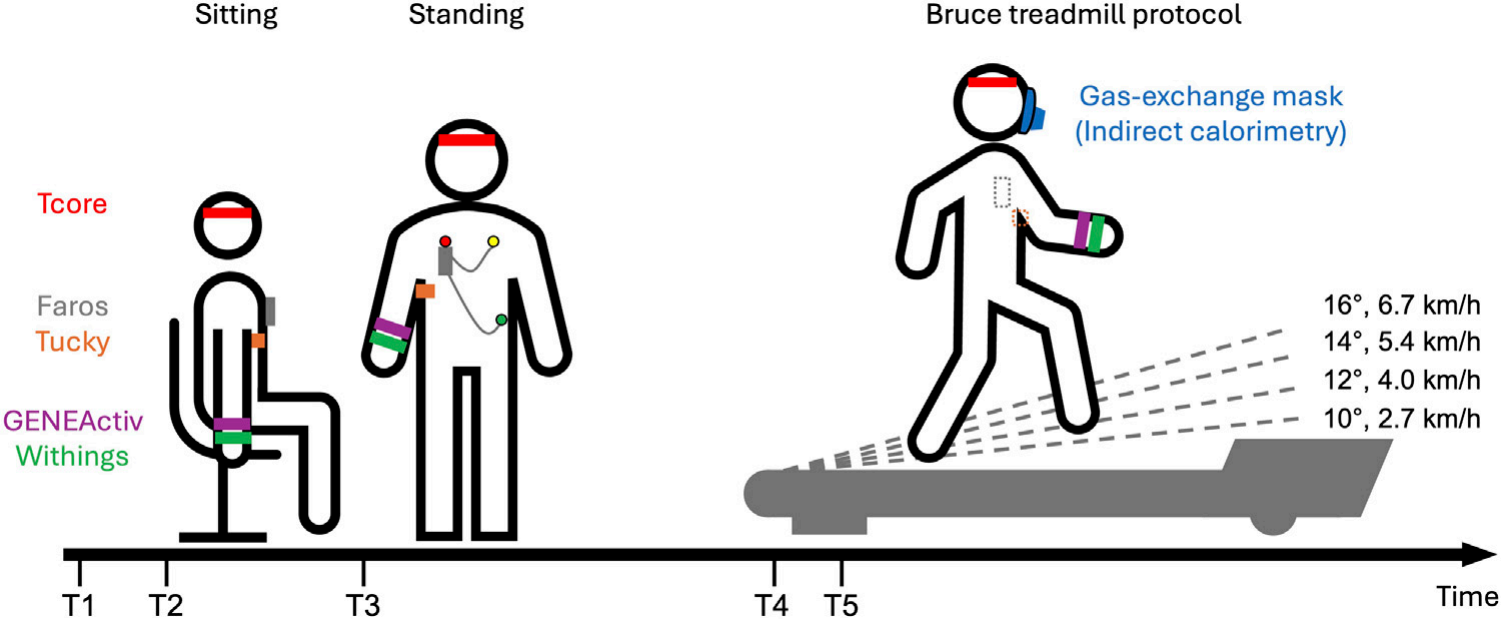
Schematic overview of the experimental protocol: Study participants were first equipped with various devices (T1). After initial setups, study participants sat for 10 min (T2) and then rested for an additioinal 10 min while standing (T3). Participants were fitted with a mask connected to the Cortex Metalyzer and rested for 3 min on the treadmill (T4). Study participants started the classic Bruce protocol (T5). The classic Bruce treadmill test consists of 3-min stages, with speed and slope increasing every 3 min without breaks. Speed and slope are displayed for the first four stages.

The measurements were conducted in the laboratories of the Institute of Physiology, Charité–Universitätsmedizin Berlin on weekdays between 9:00 and 14:30 in September 2021. Study participants followed a structured, laboratory-based protocol that included two different resting phases followed by different locomotion phases on a motorized treadmill (Figure 1). In particular, we wanted to simulate intensities typical of the daily routines of subsistence farmers in SSA regions. For example, the metabolic equivalent of task (MET) for the classic Bruce treadmill protocol is estimated to be 4.2 MET for the first stage and 8.3 MET for the third stage according to the FRIEND equation (Kokkinos et al., 2017). MET values of 4.5 and 7.8 correspond to routine chores with small animals and shovel or pitchfork work, respectively (Pickett et al., 2015).

Participants were first equipped with various devices. The Tucky device was placed under the right armpit using Tucky double-sided adhesive Adh21. Due to an initial detachment on the first study participant, we have since positioned the device closer to the chest and additionally secured it with medical adhesive tape. The Tcore sensor and headband was fastened on the participants’ forehead. The Faros was positioned on the chest and secured with medical adhesive tape to ensure signal quality. GENEActiv and Withings were placed on the wrist of the non-dominant arm, with GENEActiv positioned directly above Withings.

After these initial setups, participants sat for 10 min and then rested for an additional 10 min while standing. Following this period of rest, measurements continued on a motorized treadmill (h/p/cosmos quasar med 4.0, Nussdorf-Traunstein, Germany). Similar comparative studies also utilized treadmills as test environments (Thiebaud et al., 2018; Thomson et al., 2019). On the treadmill, participants were fitted with a mask over their mouth and nose which was connected to the Cortex Metalyzer, and were also fitted with a harness system to prevent falls on the treadmill. After a 3-min rest on the treadmill, the study participants started the Bruce protocol continuing until complete exhaustion. The classic Bruce treadmill test consists of 3-min stages, with speed and slope increasing every 3 minutes without breaks (Fletcher et al., 2013). The first four stages are as follows: Stage1: 2.7 km/h (1.7 mph), 10%; Stage2: 4.0 km/h (2.5 mph), 12%; Stage3: 5.4 km/h (3.4 mph), 14%; Stage4: 6.7 km/h (4.2 mph), 16%.

Following the treadmill test, the collected data were retrieved and stored on a study computer. Data from Withings and Tucky were downloaded from their respective platforms and spiroergometric data were exported via MetaSoft software, while Tcore, Faros, and GENEActiv data were transferred directly from their internal storage. To ensure synchronization among all considered data logs for later data analysis, timestamps were documented during the experiments. First, the times on the computers associated with the different monitors were recorded at the beginning of each measurement day to account for potential time offsets. This was necessary because GENEActiv, Faros and Tcore were initialized with the study computer, while Withings and Tucky were initialized with the same mobile device, and spiroergometry was conducted using a separate computer. Secondly, the time (on the study computer) at which the rest and activity measurements began was noted.

### 2.4 Analysis

#### 2.4.1 Data processing

For the data analysis, we considered the period from minute 3 to 8 (6 min) of the 10-min rest phases in sitting (Sit) and standing (Stand) to reduce variability due to excitement or changes in posture. Only the first four stages of the Bruce protocol were analyzed, as all 22 study participants successfully completed these stages. Recordings required processing due to differing units and sampling rate. Faros’ R-R intervals were transformed to HR (using the formula: HR = 60/R-R) and synchronized with the HR measurements taken every second by the Withings wearable. Both HR_Faros_ and HR_Withings_ were then averaged to 1-min intervals. Tucky measures temperature under the armpit (axillary temperature). To obtain an equivalent rectal (core body, CBT) temperature and enable comparison with Tcore temperature (CBT_Tcore_), we added 0.7°C to the recorded Tucky temperature (CBT_Tucky_) as suggested by the Tucky sensor description. CBT_Tcore_, initially recorded at 0.5 Hz, was averaged to 1-min intervals. The step count estimate from Withings (SC_Withings_) was compared with SC_GENEactiv_, the result of a step counting function implemented in the R package “GENEAclassify” (Campbell et al., 2023). The input for this function was the vector magnitude, VM = sqrt (x^2^+y^2^+z^2^), which we calculated from the tri-axial acceleration data recorded with the GENEActiv. Since the GENEActiv sampling rate was initially set to 10 Hz to be consistent with in field studies, SC_GENEActiv_ was averaged to 1-min intervals. Energy expenditure during the Bruce test was captured using three different approaches. The first was the indirect calorimetry method, the gold standard for determining energy expenditure by measuring the volume of oxygen consumed and the volume of carbon dioxide produced (Ndahimana and Kim, 2017). Output of indirect calorimetry (EE_IC_) was the objective measure of the metabolic equivalent of task (MET, 1MET = 3.5 mlO_2_ kg^−1^ min^−1^). The second was with Withings (EE_Withings_), which however provide data values in kcal per minute. We converted this data into MET using an equation presented in ACSM’s Guideline for Exercise Testing and Prescription (Riebe, 2014). In the third approach, EE was estimated with a prediction formula (EE = 5.01 + 1.000 ENMO) derived from accelerometry data (EE_GENEActiv_) of free-living adults (White et al., 2016). We calculated the Euclidian norm minus one (ENMO = VM-1) again using the tri-axial acceleration data recorded with the GENEActiv.

#### 2.4.2 Statistical analysis

For the resting (Sit, Stand) and locomotion phases (Stage1, Stage2, Stage3, and Stage4), agreement between two approaches was verified using the following indicators to facilitate comparison with related previous works.

• Pearson correlation: This coefficient *r* was determined to specify the degree of linear relationship.
• Lin’s concordance correlation coefficient (LCCC): Lin’s CCC includes precision in addition to Pearson’s *r* (Lin, 1989), providing a more comprehensive measurement of agreement.
• Bland–Altman method (Bland and Altman, 1986): This method provided the mean difference between the methods (bias) and the limits of agreement (LoA, bias±1.96SD of the differences). Lin’s CCC and Bland-Altman analysis were carried out with the R package “SimplyAgree” (Caldwell, 2022).
• Mean absolute percentage error (MAPE): MAPE was calculated according to the formula:

$$MAPE=\frac{100}{n}\sum_{t=1}^{n}\left|\frac{{CG}_{t}-{RG}_{t}}{{CG}_{t}}\right|$$

where CG_t_ represented the consumer-grade measurement and RG_t_ represented the research-grade measurement.

The difference between two methods was tested using the *t*-test or the Wilcoxon signed-rank test, depending on the result of the Shapiro-Wilk test for normality. The level of significance was set at 0.05 (two-sided), and *P* values were adjusted according to Holm to account for multiple testing. All statistical analyses were carried out using R (version 4.2.0; R Core Team, 2022). Scatterplots and bar charts were created with the R package “ggplot2” (Wickham, 2016).

## 3 Results

The final dataset for HR, CBT, SC, and EE analysis included 21 participants. To ensure data quality, we excluded HR data of one participant, as 40% of the HR_Withing_ readings during Sit, Stand, and Stage1 were between 43 and 58 bpm, inconsistent with the non-athlete status of our study participants. Additionally, the associated HR_Faros_ readings were almost twice as high each time. For CBT, data from one participant were excluded because the Tucky wearable fell off during treadmill exercise. For SC and EE, one GENEActiv file was corrupted.

**FIGURE 2:**
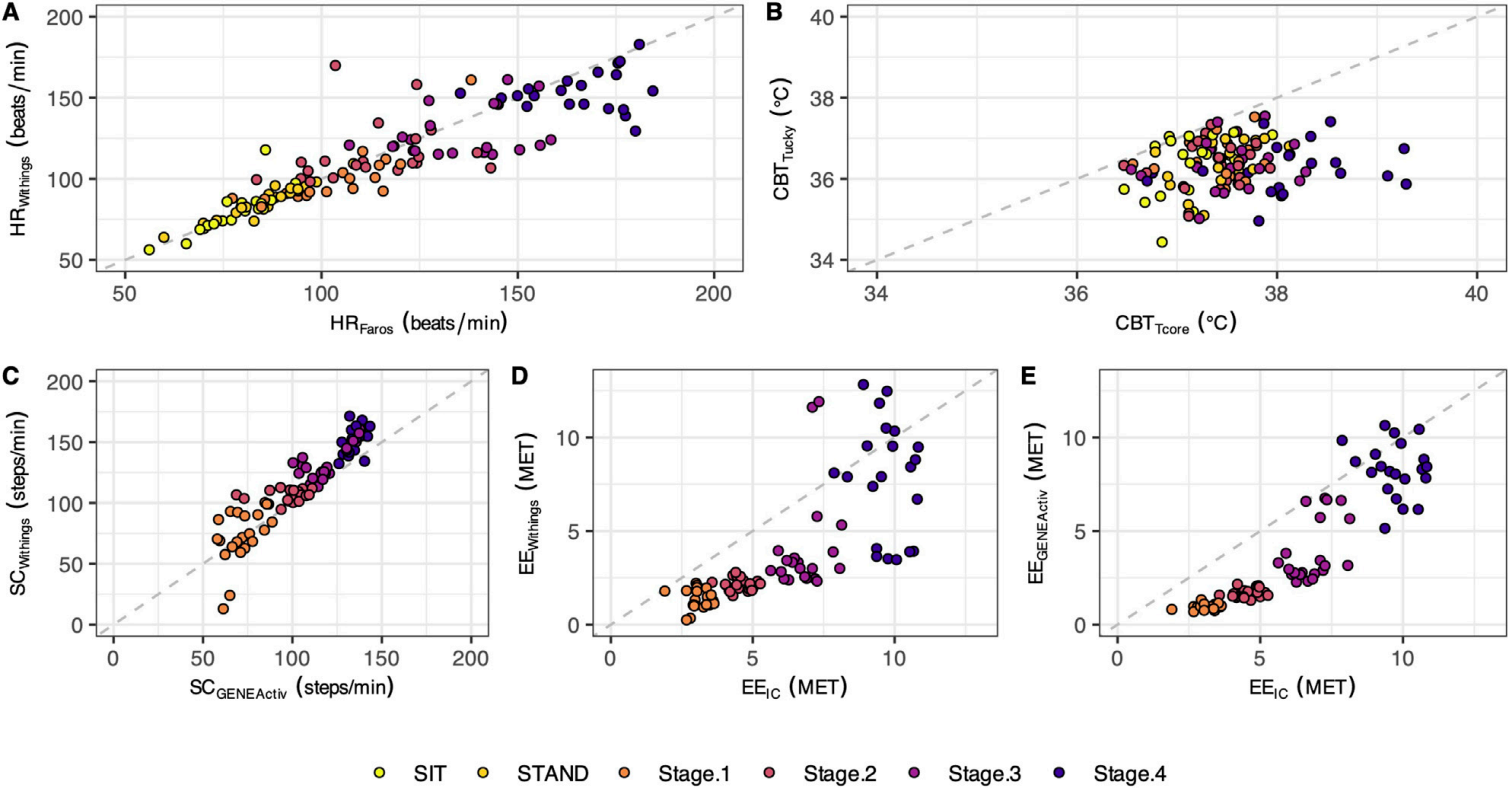
Scatterplots with the identity line. The plotted points represent individual mean values (n = 21) for the different test phases (each phase shown in a different color). The scatterplots illustrate the data for heart rate **(A)**, core body temperature **(B)**, step count **(C)**, and energy expenditure **(D, E)**.

**FIGURE 3:**
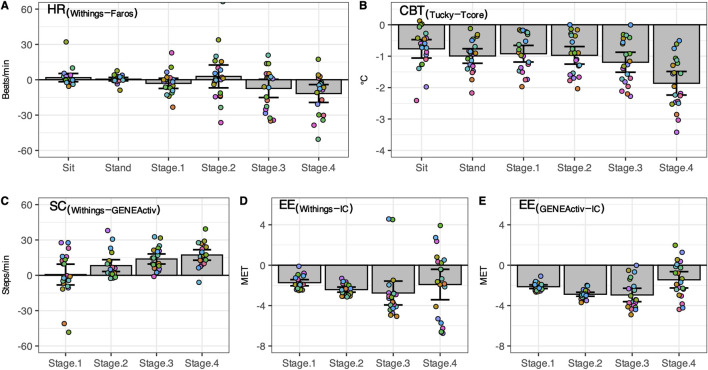
Difference between consumer-level and research-grade monitors. Individual differences (n = 21, circles) as well as mean ± 95% CI are shown for heart rate **(A)**, core body temperature **(B)**, step count **(C)**, and energy expenditure **(D, E)**. Sit, sitting position; Stand, standing position; Stage1 to Stage4, first four stages of the classic Bruce treadmill test; IC, indirect calorimetry.

Figure 2 displays scatterplots comparing HR, CBT, SC, and EE across all phases. Individual differences between methods are shown in Figure 3. Table 2 provides an overview of HR, CBT, SC, and EE values during rest and locomotion phases, including statistical summaries. Table 3 summarizes the agreements between the methods for all phases.

**TABLE 2:** Summary of heart rate, core body temperature, step count, and energy expenditure during rest and locomotion phases measured using a consumer-grade and a research-grade method (n = 21).

Variable and condition	Consumer-grade^a^	Research-grade^a^	Consumer minus research		
			95% CI	*P* value	*P* value_adj_
HR					
Sit	80.5 (13.2)	78.7 (9.3)	−1.7 to 5.3	0.97^b^	0.99
Stand	86.6 (10.3)	86.1 (10.5)	−1.0 to 2.0	0.49	0.99
Stage1	100.4 (17.5	103.5 (15.0)	−7.4 to 1.2	0.15	0.62
Stage2	115.7 (18.7)	112.8 (15.7)	−6.9 to 12.5	0.55	0.99
Stage3	128.2 (15.1)	136.3 (14.2)	−15.1 to 0.3	0.06	0.31
Stage4	154.0 (12.4)	165.7 (13.2)	−19.3 to −4.1	0.004	0.026
CBT					
Sit	36.4 (0.8)	37.2 (0.4)	−1.1 to −0.5	<0.001	<0.001
Stand	36.4 (0.5)	37.4 (0.3)	−1.2 to −0.8	<0.001	<0.001
Stage1	36.5 (0.6)	37.4 (0.3)	−1.2 to −0.6	<0.001	<0.001
Stage2	36.4 (0.6)	37.4 (0.4)	−1.3 to −0.7	<0.001	<0.001
Stage3	36.4 (0.6)	37.5 (0.4)	−1.5 to −0.9	<0.001	<0.001
Stage4	36.3 (0.6)	38.1 (0.7)	−2.3 to −1.5	<0.001	<0.001
SC					
Stage1	72.2 (22.1)	71.5 (9.4)	−8.2 to 9.4	0.61^b^	0.61
Stage2	106.3 (5.2)	98.1 (11.4)	3.2 to 13.2	<0.001^b^	0.001
Stage3	131.9 (13.5)	118.0 (11.3)	9.6 to 18.2	<0.001	<0.001
Stage4	152.2 (11.1)	134.9 (4.8)	1.28 to 21.8	<0.001	<0.001
EE_1_					
Stage1	1.3 (0.5)	3.1 (0.4)	−2.0 to −1.4	<0.001^b^	<0.001
Stage2	2.1 (0.3)	4.6 (0.4)	−2.7 to −2.1	<0.001	<0.001
Stage3	4.1 (2.7)	6.8 (0.7)	−4.0 to −1.6	0.004^b^	0.008
Stage4	7.8 (3.1)	9.7 (0.8)	−3.4 to −0.4	0.015	0.015
EE_2_					
Stage1	1.0 (0.2)	3.1 (0.4)	−2.3 to −1.9	<0.001	<0.001
Stage2	1.7 (0.2)	4.6 (0.4)	−3.1 to −2.7	<0.001	<0.001
Stage3	3.9 (1.7)	6.8 (0.7)	−3.6 to −2.2	<0.001^b^	<0.001
Stage4	8.3 (1.5)	9.7 (0.8)	−2.2 to −0.6	0.001	0.004

Variable: HR, heart rate (bpm) of Withings and Faros; CBT, core body temperature (°C) of Tucky and Tcore; SC, step count (steps/min) of Withings and GENEActiv; EE_1_, energy expenditure (MET) of Withings and indirect calorimetry; EE_2_, energy expenditure (MET) of GENEActiv and indirect calorimetry. Condition: Sit, sitting position; Stand, standing position; Stage1 to Stage4, first four stages of the classic Bruce treadmill test. P value_adj_: P value corrected for multiple comparison.

^a^ Mean (SD).

^b^ Wilcoxon signed-rank test (otherwise *t*-test).

**TABLE 3:** Relationship and agreement between the methods for heart rate, core body temperature, step count, and energy expenditure during rest and locomotion phases (n = 21).

Variable and condition	*r*	LCCC	LoA^a^	MAPE (%)
HR				
Sit	0.82	0.76	1.8 (15.1)	4
Stand	0.95	0.95	0.5 (6.3)	3
Stage1	0.84	0.81	−3.1 (18.6)	7
Stage2	0.24	0.23	2.8 (41.8)	12
Stage3	0.33	0.29	−7.4 (33.3)	11
Stage4	0.16	0.11	−11.7 (32.7)	10
CBT				
Sit	0.53	0.22	−0.8 (1.3)	2
Stand	0.40	0.10	−1.0 (1.0)	3
Stage1	0.23	0.07	−0.9 (1.1)	3
Stage2	0.23	0.07	−1.0 (1.2)	3
Stage3	0.17	0.04	−1.2 (1.4)	3
Stage4	0.19	0.04	−1.8 (1.6)	5
SC				
Stage1	0.48	0.35	0.6 (38.0)	38
Stage2	0.30	0.16	8.2 (21.6)	8
Stage3	0.73	0.43	13.9 (18.4)	10
Stage4	0.48	0.11	17.3 (19.2)	11
EE_1_				
Stage1	−0.02	0.00	−1.7 (1.3)	200
Stage2	−0.09	0.00	−2.4 (1.1)	118
Stage3	0.29	0.07	−2.8 (5.1)	113
Stage4	−0.19	−0.07	−1.9 (6.5)	60
EE_2_				
Stage1	0.16	0.00	−2.1 (0.8)	228
Stage2	0.25	0.01	−2.9 (0.9)	176
Stage3	0.46	0.09	−2.9 (2.9)	100
Stage4	−0.16	−0.07	−1.4 (3.5)	26

Variable: HR, heart rate (bpm) of Withings and Faros; CBT, core body temperature (°C) of Tucky and Tcore; SC, step count (steps/min) of Withings and GENEActiv; EE_1_, energy expenditure (MET) of Withings and indirect calorimetry; EE_2_, energy expenditure (MET) of GENEActiv and indirect calorimetry. Condition: Sit, sitting position; Stand, standing position; Stage1 to Stage4, first four stages of the classic Bruce treadmill test. LCCC, Lin’s concordance correlation coefficient; MAPE, mean absolute percentage error.

^a^ LoA: limits of agreement, bias (1.96SD).

### 3.1 Heart rate

In both resting states, the heart rate was similar for both methods. With increasing physical activity, HR_Withings_ did not increase to the same extent as the HR_Faros_ (Figure 3A). At the 4th stage, the mean difference between the methods was −12 bpm, the largest and statistically significant (Table 2). Correlations were strong and positive for Sit and Stand (*r* ≥ 0.82, LCCC ≥ 0.76). However, the agreement between HR_Withings_ and HR_Faros_ decreased with increasing physical activity (Table 3). For example, MAPE was more than twice as high from Stage2 (≥10%) as during both resting phases (≤4%).

### 3.2 Core body temperature

CBT_Tucky_ was consistently lower than CBT_Tcore_ in all phases (Figure 2B), which was confirmed by statistical analysis (Table 2). The difference between the methods was smallest at rest (Sit, −0.8°C, *t*_20_ = −5.44, *P* < 0.001) and largest in the fourth stage of the Bruce test (−1.8°C, *t*_20_ = −10.35, *P* < 0.001). CBT_Tucky_ remained unchanged across different situations (ranged between 36.3°C and 36.5°C), while CBT_Tcore_ increased with physical effort (ranging between 37.2°C and 38.1°C). Similar to HR, the correlations between the temperature monitors declined with physical activity. In addition, LoA became wider and the MAPE increased (Table 3).

### 3.3 Step count

At a treadmill speed of 2.7 km/h (Stage1), step counts were similar between SC_Withings_ and SC_GENEActiv_ (72.2 vs. 71.5 steps/min, z = 0.54, *P* = 0.61). However, the difference between the methods increased with increasing speed (Figure 3C), while SC_Withings_ increasingly exceeding SC_GENEActiv_ (Table 2). For example, at a treadmill speed of 6.7 km/h (Stage4), SC_Withings_ exceeded SC_GENEActiv_ by about 17 steps/min. (152.2 vs. 134.9 steps/min, *t*_20_ = 8.07, *P* < 0.001). On the other hand, LoA at Stage4 was only half as wide as at Stage1 (Table 3). MAPE was highest in Stage1 (38%), but was only around 10% in the following three stages.

### 3.4 Energy expenditure

EE_IC_ increased with each subsequent intensity level of the Bruce test (3.1, 4.6, 6.8 and 9.7 MET for Stage1 to Stage4). Reference EE_IC_ was significantly underestimated by both alternative methods, EE_Withings_ and EE_GENEActiv,_ in each of the four treadmill stages (Figures 3D,E). The bias to IC increased for both methods during the first three stages (up to −2.9 MET). At Stage4, the bias was only −1.9 MET (EE_Withings_) and −1.4 MET (EE_GENEActiv_), but the LoA was widest at this stage. Although the agreement between EE_GENEActiv_ and EE_IC_ appeared to be better than between EE_Withings_ and EE_IC_, the agreement between the methods for EE was generally low (Table 3).

## 4 Discussion

In this study, we measured HR, CBT, SC, and EE during both rest and treadmill phases using reference methods and consumer-grade devices (Withings Pulse HR and Tucky thermometer). We evaluated the accuracy of these parameters against established reference methods (Faros for HR, Tcore for CBT, GENEActiv for SC, and indirect calorimetry for EE). Our results showed that the wrist-worn Withings wearable demonstrated poor agreement or significant differences compared to Faros for HR, indirect calorimetry for EE, and to step-count method using tri-axial acceleration data from GENEActiv. The agreement between Tucky’s rectal equivalent and Tcore’s CBT was low at rest and during the treadmill test with significant temperature differences ranging from −1.8 to −0.8°C.

### 4.1 Comparison with previous work

In a previous validation study of wearables for HR measurement, a LCCC>0.80 was presented as an acceptable accuracy (Gillinov et al., 2017). Accordingly, our results showed that the Withings device demonstrated acceptable agreement with Faros for low physical activities (Sit: LCCC = 0.76, Stand: LCCC = 0.91, Stage1: LCCC = 0.81). The same applies if MAPE threshold is less than 10% (Boudreaux et al., 2018). In our study, MAPE was ≤4% during both resting states and ranged between 7 and 12% during the treadmill locomotion. However, in another study, the device under test was only considered valid if several criteria were met, e.g., LCCC>0.90 and MAPE<5% (Navalta et al., 2020). Furthermore, agreement in HR with the criterion measure during physical activity seems to be lower than during rest, which is in line with previous findings (Thomson et al., 2019; Bent et al., 2020). Devices that use photoplethysmography to monitor HR tend to be inaccurate at higher intensities of physical activity due to artifacts caused by intense hand movements (Castaneda et al., 2018; Bent et al., 2020; Navalta et al., 2020). In addition to motion artefacts from physical activity, ambient light, misalignment between the skin surface, and poor tissue perfusion can also be a source of error (Alzahrani et al., 2015). Skin tone is apparently not a source of errors (Bent et al., 2020), which is an important observation for studies involving African populations, for example. Interestingly, Stahl et al. (2016) observed a decrease in MAPE at treadmill speeds of >3.2 km/h, attributing this to improved perfusion due to increased intensity. In the present study, a small decline in MAPE was observed at treadmill speeds of >4.0 km/h. Nevertheless, not only the user of wrist-worn HR monitor or the ambient conditions seem to affect measurement accuracy, but also the device itself. Müller et al. (2019) investigated the validity of HR measures of a high-cost consumer-based tracker and a low-cost tracker in a laboratory setting, showing the high-cost tracker had smaller errors and a higher agreement with the criterion measure than the low-cost tracker.

For a step counter to be considered accurate, the MAPE should be less than 1% compared to the criterion measure when walking on a treadmill at a speed of 4.8 km/h (Tudor-Locke et al., 2006). In a recent review of the validation of treadmill step-counting technologies, median MAPE values for wrist-worn monitors ranged from 6.6% to 10.7% at speeds between 3.2 and 6.4 km/h (Moore et al., 2020). In our study, the MAPE ranged from 8% to 38% at speeds between 2.7 and 6.7 km/h (Stage1 to Stage4). In addition, the bias was lowest for Stage1 at 0.6 steps/min and highest for Stage4 at 17.3 steps/min, indicating an increasing overestimation in steps by the Withings Pulse HR with increasing treadmill speed. On the other hand, one could argue that estimating steps using a step counting algorithm with tri-axial acceleration data is not a gold standard. Therefore, we compared the estimates in our study with published hand-counted steps from treadmill experiments of Ducharme et al. (2021) and Tudor-Locke et al. (2019) (Supplementary Table S1). It was shown that both the SC_Withings_ and the SC_GENEActiv_ estimated about 17 steps/min less at speed of 2.7 km/h, which was the largest difference compared to published data. Low accuracy of step counting at slow walking speeds is a common issue with wrist-worn wearables (Moore et al., 2020). At treadmill speeds of 5.4 and 6.7 km/h, differences between hand-count SC_Withings_ were about −12 and −18 steps/min, while differences between hand-count and SC_GENEActiv_ were only about 2 and -1 steps/min. These observations suggest a paradox: bias was best at slow walking speed of 2.7 km/h because both wearables were equally inaccurate. Since the use of raw acceleration data provides a flexibility in processing, selecting a better performing step count function should be considered. For example, Ducharme et al. (2021) recently published a transparent algorithm for step detection, and the open-source Verisense step count algorithm has been optimized (Maylor et al., 2022; Rowlands et al., 2022). While Withings Pulse HR utilizes changes in the acceleration caused by foot impact during walking, the exact algorithm is not disclosed.

The EE_Withings_ showed low overall agreement with EE_IC_ during the treadmill test. The same applies to EE_GENEActiv_, where acceleration data from GENEActiv was used to estimate EE using a prediction formula for physical activity energy expenditure (White et al., 2016). In both comparisons, the MAPE value was very high at Stage1 (≥200%), but decreased with increasing treadmill locomotion levels and was lowest in Stage4 (Withings: 60%, GENEActiv: 26%). However, Passler et al. (2019) considered a tested device valid if MAPE is less than 10%. The decrease in MAPE with increasing treadmill speed (and grade) indicates better agreement with higher physical workload. In fact, estimated HR by wrist-worn photoplethysmography devices in combination with physiological modeling tended to have lower MAPE for EE estimation during activities above the aerobic threshold (Parak et al., 2017). Moreover, in the present study both wrist-worn devices for EE estimation clearly underestimated the EE for the criterion measure (indirect calorimetry). Wearable trackers for EE estimation predominantly underestimate EE even in a controlled environment (Evenson et al., 2015; Wahl et al., 2017; Fuller et al., 2020). Wearables were typically examined while worn on the wrist (Fuller et al., 2020), though a greater accuracy can be achieved when placed on the hip or shirt collar (Woodman et al., 2017). EE estimates from devices worn on the wrist or hip generally vary in accuracy depending on physical intensity and type of activity (Howe et al., 2009; O’Driscoll et al., 2020). Recently, Ogata et al. presented an equation to improve EE estimation using accelerometer-based MET value and individual HR and showed that estimated total energy expenditure in rescue workers was one-third higher with the combined approach than with the accelerometer-based method alone (Ogata et al., 2024).

Most wearable thermometers were developed to continuously monitor skin temperature, few in order to reflect changes in CBT (Tamura et al., 2018). In the present study, we compared two sensors attached to the skin: the Tucky thermometer under the right armpit and the Tcore sensor on the forehead. Although adding 0.7°C to the measured values of Tucky improved agreement with rectal temperature, correlations between Tucky’s rectal measurements and Tcore’s CBT estimate decreased with increased physical activity (highest during Sit and the lowest during Stage4 of the Bruce treadmill test). In addition, the bias in each of the six activity phases was at least −0.8°C, indicating that Tucky’s rectal measurements underestimated traditional rectal temperature measurement. For example, Gunga et al. (2008) validated the Tcore precursor with rectal temperature measurement during treadmill activities (25%–55% maximum work intensity) at different ambient temperatures, demonstrating a good agreement during resting (Bias: 0.01°C, LoA: 0.74 to 0.72) and working periods (Bias: 0.08°C, LoA: 0.77 to 0.61) at ambient temperature of 25°C. Wearable thermometers are considered in agreement if they comply with the clinically meaningful recommendations of bias of ±0.5°C and LoA of ±1.0°C (Tamura et al., 2018). In our study, however, the bias was at least −0.8°C and the LoA were −2 to 0°C at Stand (narrowest) and −3.5°C to 0.2°C at Stage4 (widest). Our results suggest that the higher the intensity of physical activity, the lower the accuracy of Tucky’s measurements. This inaccuracy could be attributed to the thermoregulatory processes of the skin. Increased physical activity can lead to increased perspiration, which aims to cool the skin and CBT through evaporation. In the context of varying and intensive physical activity, Tucky under the armpit did not achieve sufficient accuracy with CBT. Similar observation was reported for another adesive axillary thermomenter patch. Temperatures of adesive axillary thermomenter showed good agreement with those from the conventional axillary method (Bias: 0.15°C, LoA: 1.13 to 0.99), but failed to those of the bladder as the CBT (Bias: 1.11°C, LoA: 3.19 to 0.98) (Boyer et al., 2021).

### 4.2 Strength and limitations

This study has several strengths. Firstly, we investigated two devices, Withings Pulse HR and Tucky thermometer, that had not been validated in an independent lab study previously, focusing on their utility for in-field assessment of physiological variables in different situations of varying physical activity. Therefore, a structured protocol consisting of successively changing intensities of activity was implemented. A structured procedure and laboratory-based setting enabled a high precision of comparison and reproducibility of results.

This study was limited to healthy, fair-skinned adults aged 20–29 years. Future research should include a more diverse cohort and a comparison of multiple skin tones, especially when using optical heart rate monitors. Motion that largely affects positioning of wearables, such as treadmill running for a wrist-worn tracker, may impact accuracy and the significance of validation research. Potential interference between devices worn simultaneously on the same wrist might also represent a possible limitation of the study. Additionally, although treadmill-based incremental testing can represent the cardiovascular strain of physical activity during agricultural work, it does not correspond to the actual biomechanics and motions of such physical activity.

## 5 Conclusion

In recent years, research interest in consumer-grade wearables has surged, driven by the potential of these sensors for a broad range of applications, from on-the-field ergonomic assessments to follow-ups in rehabilitation medicine. In this study, we evaluated the Withings Pulse HR wearable for HR, SC, and EE quantification and the Tucky thermometer for CBT. The Withings device demonstrated good performance in HR monitoring at low physical activity intensities and in SC at higher activity levels. However, the agreement between the Tucky thermometer measured temperature and CBT was low at rest and gradually declined with increased physical activity. In summary, both evaluated consumer-grade wearables did not achieve adequate accuracy for research purposes in controlled environments. However, Withings Pulse HR may be useful for long-term monitoring in the field, as it can effectively detect and recognize general changes in activity and corresponding physiological variables (HR, SC, EE) despite its lack of precision.

## Data Availability

The raw data supporting the conclusions of this article will be made available by the authors, without undue reservation.
