# Supplementary material for: Laboratory comparison of consumer-grade and research-established wearables for monitoring heart rate, body temperature, and physical acitivity in sub-Saharan Africa
Source: Front Physiol. 2025 Feb 13;16:1491401. doi: 10.3389/fphys.2025.1491401 (PMC11865084; doi:10.3389/fphys.2025.1491401)
Supplement: Supplementary file 1 [file Table1.docx]

Supplementary Material

# Supplementary Table

Table S1. Average steps per min at different treadmill speed.

| Treadmill Speed | | |  | hand-counted | |  | present study | |  |
| --- | --- | --- | --- | --- | --- | --- | --- | --- | --- |
| km/h | mph |  | | Tudor-Locke | Ducharme |  | SC_GENEActiv_ | SC_Withings_ |  |
| 2.4 | 1.5 |  | | 83.8 | 83.9 |  |  |  |  |
| 2.7 | 1.7 |  | | **88.7** | **88.9** |  | 71.5 | 72.2 |  |
| 3.2 | 2.0 |  | | 96.1 | 96.4 |  |  |  |  |
| 4.0 | 2.5 |  | | 105.8 | 106.2 |  | 98.1 | 106.3 |  |
| 4.8 | 3.0 |  | | 113.6 | 113.9 |  |  |  |  |
| 5.4 | 3.4 |  | | **119.9** | **120.1** |  | 118.0 | 131.9 |  |
| 5.6 | 3.5 |  | | 121.5 | 121.7 |  |  |  |  |
| 6.4 | 4.0 |  | | 129.0 | 129.2 |  |  |  |  |
| 6.7 | 4.2 |  | | **133.4** | **134.0** |  | 134.9 | 152.2 |  |
| 7.2 | 4.5 |  | | 139.9 | 141.3 |  |  |  |  |
| Hand-counted steps are from treadmill tests at different speeds and without slopes from the studies of Tudor-Locke et al. (2019) and Ducharme et al. (2021). In contrast, we estimated the steps during the standard Bruce treadmill test (different speeds and slops). To enable a comparison between hand-counted and the presented estimated steps, the missing hand-counted steps (bold values) were linearly interpolated using the neighboring values as start and end points. | | | | | | | | | |

Ducharme, S. W., Lim, J., Busa, M. A., Aguiar, E. J., Moore, C. C., Schuna, J. M., et al. (2021). A Transparent Method for Step Detection using an Acceleration Threshold. *J Meas Phys Behav* 4, 311–320. doi: 10.1123/jmpb.2021-0011

Tudor-Locke, C., Aguiar, E. J., Han, H., Ducharme, S. W., Schuna, J. M., Barreira, T. V., et al. (2019). Walking cadence (steps/min) and intensity in 21–40 year olds: CADENCE-adults. *International Journal of Behavioral Nutrition and Physical Activity* 16, 8. doi: 10.1186/s12966-019-0769-6
